# Proteomic Responses of the Springtail *Folsomia candida* to Drought

**DOI:** 10.3390/insects16070707

**Published:** 2025-07-09

**Authors:** Yang Wang, Stine Slotsbo, Steffen Y. Bak, Christopher J. Martyniuk, Martin Holmstrup

**Affiliations:** 1 Department of Ecoscience, Aarhus University, C.F. Møllers Allé 4, 8000 Aarhus, Denmark; wybiology351@gmail.com (Y.W.); stsl@ecos.au.dk (S.S.); 2 International Flavors & Fragrances Inc., Edwin Rahrs Vej 38, 8220 Brabrand, Denmark; steffen.yde.bak@iff.com; 3 Center for Environmental and Human Toxicology, University of Florida, 2187 Mowry Road, Gainesville, FL 32611, USA; cmartyn@ufl.edu

**Keywords:** Collembola, desiccation, protein regulation, pathway analysis, metabolism

## Abstract

Springtails are adapted to life in the pore space of soil, where humidity in moist soil is close to saturation. Drought is the most important limiting factor for springtails; however, their molecular and physiological adaptations to low humidity are not well understood. The present study explored the global proteomic drought response of the springtail, *Folsomia candida* (Isotomidae, Collembola). In relatively dry soil (−360 kPa), adult springtails initially lost body water but re-established the normal body water content over the following two weeks. Nano LC–MS/MS analysis identified a total of 1729 unique proteins. Proteomic analysis and pathway enrichment found that the proteome generally did not show a dramatic induction of proteins in response to drought stress. After an initial down-regulation of pathways related to metabolism and growth, these pathways gradually returned to the same levels as in moist soil. Other pathways such as the cytoskeleton pathway, which is important in cell proliferation and differentiation, were predominantly down-regulated throughout the experiment in drought-exposed animals, which correlated with essentially no somatic growth of the springtails in dry soil. This study facilitates the understanding of the consequences of climate change on soil functioning and fertility.

## Simple Summary

Springtails are small insect-like animals living in the soil almost all over the world. Most springtails are less than 5 mm in length, yet they can have great influence on the fertility of soil because they occur in very high numbers, sometimes in hundreds of thousands per square meter. These animals are adapted to life in moist soil and in many respects have a physiology resembling freshwater animals. Here, we explore the functional proteins in springtails when they are stressed by drought conditions. We find that proteins important for growth and reproduction are becoming less abundant in drought-exposed animals, but on the other hand, we see no strong indication of proteins related to cellular stress handling being more abundant. This suggests that springtails will stop growing and egg laying when they are experiencing drought but will be able to survive until soils become moist again.

## 1. Introduction

Springtails are small arthropods living in the soil and leaf litter of most regions of the world [1]. These animals mainly feed on fungal hyphae and bacteria and, to some extent, on dead organic matter and fine roots [2,3,4]. Through their feeding habits and high population numbers, springtails can be important drivers of decomposition processes and nutrient cycling in the soil [5,6,7].

Many springtail species are adapted to life in the soil pore space where humidity is close to saturation most of the time [8]. For this reason, low soil humidity may be an important limiting factor for springtails as well as for many other soil invertebrates [9,10]. The ongoing climatic changes are predicted to escalate the occurrence of summer droughts and generally cause decreasing soil moisture levels on a wide geographical scale [11,12]. Decreasing soil moisture will negatively impact the population dynamics and feeding activities of soil animals, which in turn erodes important decomposition processes in the soil.

The growth, development, and reproduction of springtails are highly dependent on sufficient humidity of the soil, and most species have optimal conditions when soils are close to the field capacity where free water is readily available for these animals [13,14,15]. Despite the apparent dependence on high soil humidity for optimal performance, springtails can osmoregulate and endure extreme drought in the soil by accumulation of compatible osmolytes such as sugars and sugar alcohols in body fluids [16,17,18]. This enables them to absorb water vapor from pore air and maintain water balance at soil humidities even below the wilting point of plants (−1.5 MPa) and survive drought for weeks [19,20,21].

*Folsomia candida* Willem (a soil dwelling springtail) is capable of water vapor absorption and survives extreme drought conditions in soil with water potentials equivalent to −6 MPa [18]. Recently, we have shown that *F. candida*, despite its remarkable drought resistance, is sensitive to subtle decreases in soil water potential [15]. Thus, reproduction was completely paused when soil water potential decreased from −2 kPa (~field capacity) to −20 kPa, and body growth ceased at −200 kPa. This observation was surprising since such drought levels should not cause evaporative water loss. In fact, the osmotic pressure of body fluids is equivalent to about −900 kPa, creating a water vapor gradient and a net influx of water from the soil and into the body fluids of the animal at these soil water potentials [21].

Currently, we do not know why common somatic traits such as growth and reproduction are ceasing to occur when water balance is, seemingly, not physiologically challenging. To address this question, we employed untargeted proteomic analysis of springtails exposed to moderately dry soil compared to animals subjected to optimal moisture conditions. When the animals are exposed to such environmental contrasts, changes in active peptides, proteins, enzymes and specific pathways can provide critical pieces of evidence for understanding dynamic and real-time responses to realistic drought conditions. Gene expression studies have previously provided some insight into the molecular mechanisms underlying tolerance of severe desiccation stress of springtails [22,23], but very little is known of the proteomic responses to drought [24]. The sequenced genome of *F. candida* provides abundant genetic information in public databases [25], facilitating protein identification and responsive pathway enrichments. Physiological responses can be dynamic over time, and therefore, we expected that the proteomic responses to drought during a 20-day period would capture proteomic responses as much as possible. Transcriptional responses of *F. candida* to drought and other environmental stressors usually take place almost immediately (hours), whereas proteomic and physiological responses often occur on a longer time scale (days) [23,26]. The present study aimed to identify candidate pathways to increase our understanding of the dynamic and real-time responses to realistic drought conditions in *F. candida* as a representative of soil dwelling springtails. We hypothesized that pathways involved in the accumulation of sugars and other drought-protective osmolytes would be up-regulated in *F. candida* as would be the case with general stress-related pathways (e.g., antioxidant defense mechanisms). In contrast, we expected pathways related to cellular growth and reproduction to be suppressed.

## 2. Materials and Methods

### 2.1. Test Animals

The parthenogenetic *Folsomia candida* (Collembola, Isotomidae, reference genome: ASM221717v1) originated from a laboratory culture kept at 20 °C (±1 °C) with a 12:12 h light–dark cycle. The test animals were cultured in Petri dishes with a moistened floor of plaster of Paris mixed with charcoal (8:1 *w*/*w*). The animals were fed dried baker’s yeast ad libitum. For the experiment, the springtails were age-synchronized to reduce variation, and only medium-sized adults (25 ± 3 days old) having a dry mass of ca. 40 µg were used for our study.

### 2.2. Test Soil

Soil from the top 0–20 cm layer was collected in Foulum Experimental Station, Department of Agroecology, Aarhus University, Denmark. The field from which test soil was sampled had been organically cultivated without pesticide application for more than 20 years. This soil was a loamy sand consisting of 32% coarse sand (>200 μm), 48% fine sand (20–200 μm), 9% silt (2–20 μm), 7% clay (<2 μm), and 4% organic matter (determined by loss-on-ignition), and it had a pH of 5.9. The total organic carbon content was 1.6% [15]. The soil was thoroughly homogenized, dried at 105 °C for 24 h, and sieved through a 2 mm mesh prior to use. Soil water content was adjusted to 6% (mild drought) and 20% (control) of dry mass by adding appropriate volumes of deionized water to the soil and mixing it thoroughly. None of these soil water levels cause mortality [15]. The actual soil water contents of test soils were determined by subtracting the soil mass before and after drying at 105 °C for 24 h and showed that the measured water content was 20.3 ± 0.4 g g^−1^ dry mass. Based on a previously published retention curve, the soil water potential of the test soils was −2.4 kPa (control) and −360 kPa (mild drought), respectively [15]. These two levels of soil water potential represent a realistic moisture range in European soils [8,20].

### 2.3. Drought Exposure Experiment

Springtails were exposed to the two soil humidities in 50 mL glass jars containing 30 g of soil. Thirty adult animals were added to each jar using five jars (replicates) for each soil humidity level and sampling time. A total of 20 mg of dried baker’s yeast was sprinkled on the surface of the soil as food source for the springtails, and the jars were closed with tightly fitting screw caps. During the experiment, water evaporation from the beakers was negligible because the lids were only briefly opened for aeration every 3 days, and 20 mg of dried yeast was added after 7 and 14 days. In total, 55 jars with animals were made for each soil humidity (11 sampling times × 5 replicates). Jars with springtails were destructively sampled after 1, 2, 3, 5, 8, 10, 14, 15, 16, 17, and 20 days. Previous experiments have shown that physiological responses of springtails to drought are well captured within 1–3 weeks of exposure [15,21]. On each sampling day, the soil of a replicate was gently spread out in a tray, and animals were recovered using an aspirator. Twenty animals were sampled for proteomic analysis, placed in 2 mL centrifugation tubes, and immediately stored at −80 °C until protein extraction and further analysis. The remaining ten animals in each replicate were sampled for measurement of body mass and body water content.

### 2.4. Body Mass and Body Water Content of Springtails

Body mass and body water content were measured at all sampling times in 5 replicates. The total fresh mass of ten animals of each replicate was determined using a Sartorius Micro SC 2 balance accurate to ±1 µg (Sartorius AG, Goettingen, Germany). After measuring the fresh mass, the dry mass of adults was obtained by weighing after freeze-drying for 24 h. The average fresh and dry mass per animal was calculated based on the number of adults, and the body water content was calculated as mg water mg^−1^ dry mass. The effects of soil humidity and time on the body mass and body water content of springtails were analyzed using linear regression models. Each model included day, soil humidity (control or drought), and their interaction (day × soil humidity) as predictors. Model assumptions were verified through visual inspection of residuals, and no transformations were required. Analyses were performed in R Studio 2023.12.1 Build 402.

### 2.5. Sample Preparation for Proteomics

Proteomic analysis of springtails was performed on days 1, 2, 5, 14, and 20, with 3 replicates per sampling time. Total proteins were extracted from springtails using 250 µL lysis buffer (5% sodium dodecyl sulfate (SDS), 100 mM triethylammonium bicarbonate (TEAB), and protease inhibitor (complete, EDTA-free, Roche, Basel, Switzerland)), followed by heating (95 °C for 10 min) and mixing at 900 rpm. Subsequently, homogenization was performed on a Precellys Evolution (Bertin technologies, Cergy-Pontoise, France) using 6000 rpm and a cycle scheme with 20 s ON/30 s OFF in 5 cycles. The homogenized samples were sonicated on a Biorupter Pico (Diagenode, Seraing, Belgium) water bath ultra sonicator at 4 °C for 10 min, followed by centrifugation at 14,000× *g*. Supernatants from each sample were used for protein-concentration measurement by a detergent-compatible Bradford assay (Pierce^TM^ Detergent-Compatible Bradford Assay Kit, Thermo Fischer, Waltham, MA, USA). A volume equal to 100 μg of proteins from each springtail sample was used for protein digestion. Protein digestion was performed using the S-Trap^TM^ digestion protocol (ProtiFi, Plainsboro, NJ, USA) but with a few modifications. Trypsin digestion was prolonged to overnight at 37 °C with a trypsin-to-protein ratio of 1:62.5 [27]. Peptides were dried down using a speed vacuum instrument at 35 °C. Dried peptides were resuspended in 50 µL 0.1% trifluoroacetic acid (TFA). Peptide concentrations were estimated using a peptide specific BCA kit following the manufacturer’s protocol (Pierce^TM^ Quantitative Peptide Assays, Thermo Scientific^TM^). Based on peptide concentrations, peptide samples were adjusted to reach the same concentrations. A QC sample was prepared by mixing 10 µL from each sample. All samples were transferred to LC vials and saved at 5 °C until analysis.

### 2.6. Data Acquisition

LC-MS/MS analysis was carried out using an UltiMate^TM^ 3000 RSLCnano System (Thermo Fisher Scientific, Waltham, MA, USA) interfaced to a Q Exactive^TM^ HF Hybrid Quadrupole-Orbitrap^TM^ Mass Spectrometer (Thermo Fisher Scientific, Waltham, MA, USA) as previously described by Kumar et al. [28]. The dissolved peptide samples were trapped by a 20 mm nano Viper Trap Column (Acclaim^TM^ PepMap^TM^ 100 C18, 3 µm particle size and an inner diameter of 0.075 mm) that was connected to a 400 mm analytical column (PepSep, ReproSil 3 µm C18 beads, pore diameter 120 Å, and an inner diameter of 0.075 mm). Separation was performed at a flow rate of 300 nL min^−1^ through a gradient method of a 40 min gradient of 0–41% Solvent B (100% ACN, 0.1% FA) and eluted into the Nanospray Flex Ion Source (Thermo Scientific^TM^) interfaced to the MS instrument. Data was acquired on the Q Exactive HF instrument in data-dependent MS/MS mode using HCD fragmentation. The peptide masses were measured in the MS scan by the Orbitrap with a resolution of 60,000 (FWHM) at *m*/*z* 200. The top 12 most intense ions were selected and fragmented in the quadrupole using a 1.2 Da width isolation window. MS/MS spectra were recorded in the Orbitrap with a resolution of 30,000 (FWHM) at *m*/*z* 200 followed by dynamic exclusion including an exclusion duration of 30 s and an exclusion mass tolerance width of ±10 ppm relative to masses on the list. Sample acquisition was designed with an initiation block of five injections of the QC sample followed with continuous blocks of five samples and one QC sample. All samples were randomly distributed over the blocks to avoid the change in block effects.

### 2.7. Data Analysis

The raw data was processed (smoothing, background subtraction, and centroiding) by Proteome Discoverer (Version 2.2, Thermo Scientific^TM^) and submitted to database searching against a UniProt *Folsomia candida* database containing 30,219 sequences using an in-house Mascot server. Trypsin was set as protease with a maximum of two missed cleavages acceptable. S-Carbamidomethyl cysteine was defined as a fixed modification and oxidation of methionine as variable modification. The MS/MS results were searched with a peptide ion mass tolerance of ±10 ppm and a fragment ion mass tolerance of ±0.8 Da. A percolator was used for calculating the false discovery rate (FDR), and only peptides that were identified as rank 1 peptides and with a confidence value of 1% (q < 0.01) were considered for further analysis [29].

Data analyses were performed in Expressionist v.12.0.9 (Genedata). Imported raw files were noise-filtered using a chemical noise subtraction. Chromatograms were retention-time-aligned by a pairwise alignment, filtered, and smoothed before peak detection, based on volumes. Detected peaks were subjected to isotopic clustering, and singletons were filtered out. Peak clusters were matched to the identifications from the Proteome Discoverer. Peptides were grouped based on protein identifications. Proteins were quantified based on the three most intense peptides Hi-3 [30]. Quantitative results were exported into Analyst v.12.0.9 (Genedata) for normalization, statistical filtering, and testing. Quantitative abundances were normalized by an intensity drift normalization, where intensities from QC samples were used to correct for drifting in the nano LC-MS/MS performance.

### 2.8. Differential Abundance Analysis and Pathway Enrichment of Proteomics Data

The differential abundance of proteins in the drought group compared to the control group was normalized and analyzed in Rstudio (4.2.2) with the EdgeR package [31]. Abundance ratios ≥ 1.5 were classified as up-regulated abundances, while those ≤ −1.5 were classified as down-regulated abundances [32]. Abundance ratios between −1.5 and 1.5 (excluding equal to −1.5 and ≤−1.5) were classified as stable. The fold change (FC) equated to the log_2_ of abundance ratios (log_2_FC ≤ −0.585 or ≥0.585), which was set as a cutoff for the up-regulated and down-regulated expressions of proteins, and the FC values were employed for all differential expressions in proteins in this study. The significant proteins with differential FC were based on a false discovery rate (FDR < 0.05) at each time point. The pathway enrichment approach used the package clusterProfiler 4.0 in R [33], using q value < 0.1 to correct the adjusted *p* values (FDR < 0.05). The top pathways and molecular events were sorted using the criteria of FDR (FDR < 0.05).

### 2.9. Heatmap Analysis and Two-Way ANOVA Analysis

MetaboAnalyst 6.0 was used to analyze protein abundance data in each treatment over time. For each biological replicate, relative protein abundance data were normalized using Quantile normalization, followed by Log transformation (base 10). Auto-scaling (mean-centered divided by the standard deviation of each variable) was then conducted to normalize data. Clustering was generated for the samples using mean centering and the Fast Ward algorithm (distance measure Euclidean) based on results of a two-way ANOVA with soil moisture level and sampling time as factors.

## 3. Results

### 3.1. Growth and Body Water Content

**Figure 1:**
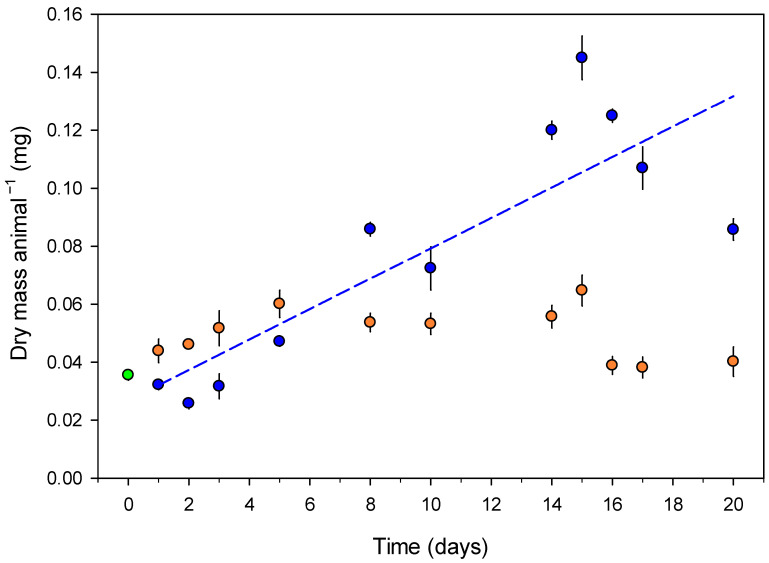
The body dry mass (mean ± SE, n = 5) of *Folsomia candida* throughout the experiment. The green dot indicates the body dry mass at the start of the experiment. Blue points and the line represent animals exposed to control soil humidity (20% of dry mass; −2.4 kPa). Orange points represent animals exposed to dry soil treatment (6% of dry mass; −360 kPa). The body dry mass increased significantly over time in the control treatment (*p* < 0.001).

Springtails exposed to control soil humidity significantly increased their dry body mass by three times during the 20-day experiment (linear regression, *p* < 0.001, Figure 1). In contrast, animals exposed to drought did not grow, and their dry mass remained stable or slightly declined over time. This was confirmed by a significant negative interaction between day and treatment in the linear model (*p* < 0.001), indicating that drought reduced growth compared to control animals. No mortality occurred during the experiment.

**Figure 2:**
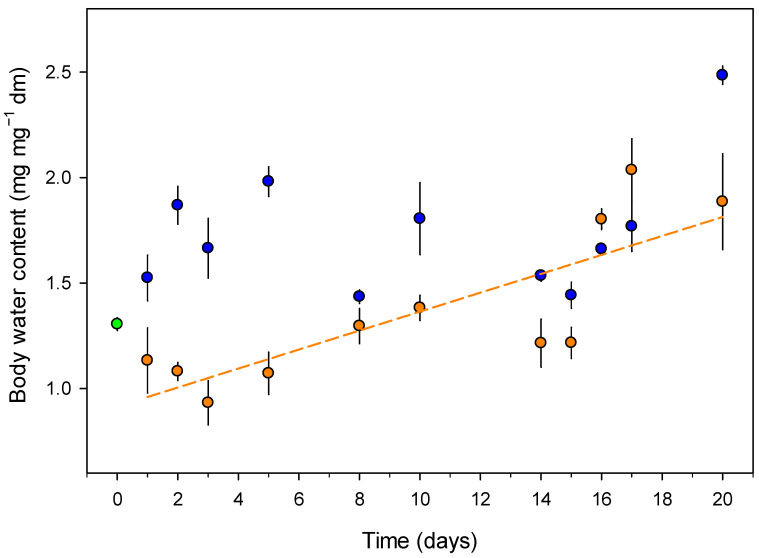
The body water content (mean ± SE, n = 5) of *Folsomia candida* throughout the experiment. The green dot indicates the initial body water content at the start of the experiment. Blue points represent animals exposed to control soil humidity (20% of dry mass; −2.4 kPa), while orange points and the line represent animals exposed to dry soil conditions (6% of dry mass; −360 kPa). Springtails exposed to dry soil initially had lower water content but increased water content more rapidly over time compared to the control group.

In the control soil, springtail body water content remained relatively stable, with a slight but non-significant increase over time (linear regression, *p* = 0.087; Figure 2). Springtails exposed to drought had significantly lower water content on day 1 compared to control animals (*p* < 0.001). However, a significant positive interaction between day and drought treatment (*p* = 0.0014) indicated that the water content in drought-exposed springtails increased more rapidly over time, eventually reaching levels similar to those of the control group by the end of the experiment.

### 3.2. Proteomics

**Figure 3:**
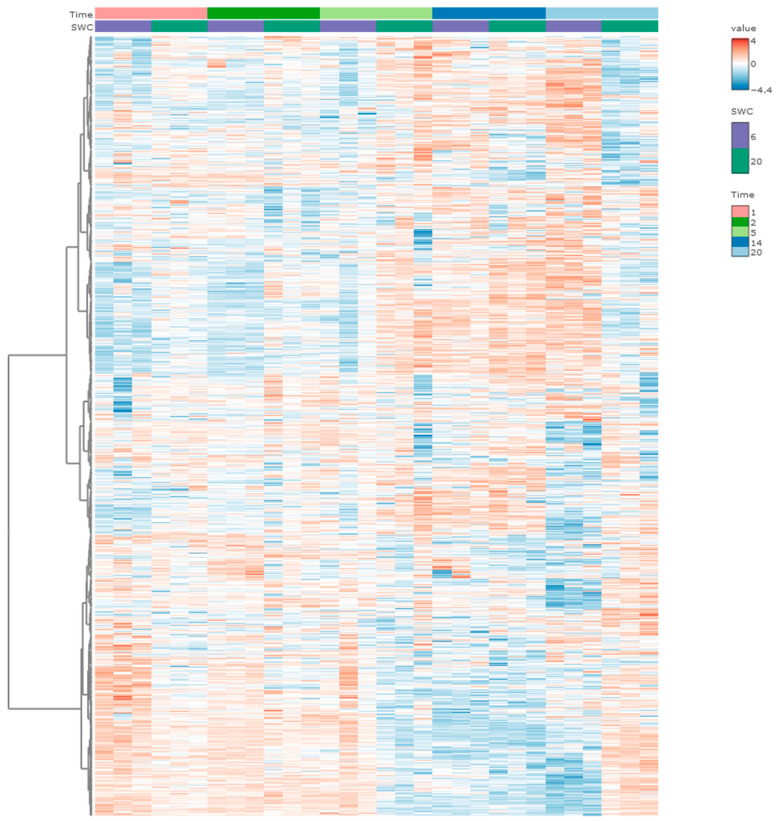
Heatmap of proteomic responses to the two soil water contents in time series. The time bar shows the days 1, 2, 5, 14, and 20 days in 5 different colors. SWC represents soil water content with two levels of 6 and 20 (percent of dry weight) using two colors. The value shows the range of fold changes of proteins in each replicate (down-regulated in blue, up-regulated in red). Each column in the heatmap is one biological replicate. n = three replicates for each treatment at each time point.

The complete dataset consisted of a total of 115 LC-MS/MS runs and was used in the protein identification and quantification workflow. In total, we identified and quantified 2315 proteins, and across all 115 runs, we had an average of 94.6% of total proteins found, providing an almost complete data matrix. After filtering to optimize the downstream analysis, a total of 1729 proteins (equivalent to ca. 75% of the identified and quantified proteins) in a clean dataset were used for further analysis. We examined the distribution of the abundances and qualities of identified proteins (Supplementary Figure S1). In the heatmap, the changes in all protein abundances have been shown with all replicates at both levels of soil water content (Figure 3). The trends of changes in protein abundances have been mainly divided into two clusters, including a dominant down-to-up trend from day 1 to 14 and, conversely, a dominant up-to-down trend from day 1 to 14 across both SWC groups (Figure 3). Among the two clusters, the protein abundances in the drought group show major responses to drought stress, including down-regulating and up-regulating proteins, compared to the control group on day 20 (Figure 3). A total of 42 proteins were significantly impacted by the factor of soil water content and, twice as many as that, 91 proteins were significantly impacted by the interaction effect of soil water content and duration of drought exposure (Supplementary Figure S2), suggesting the proteomic responses of *F. candida* attributable to soil water content levels and exposure duration.

**Figure 4:**
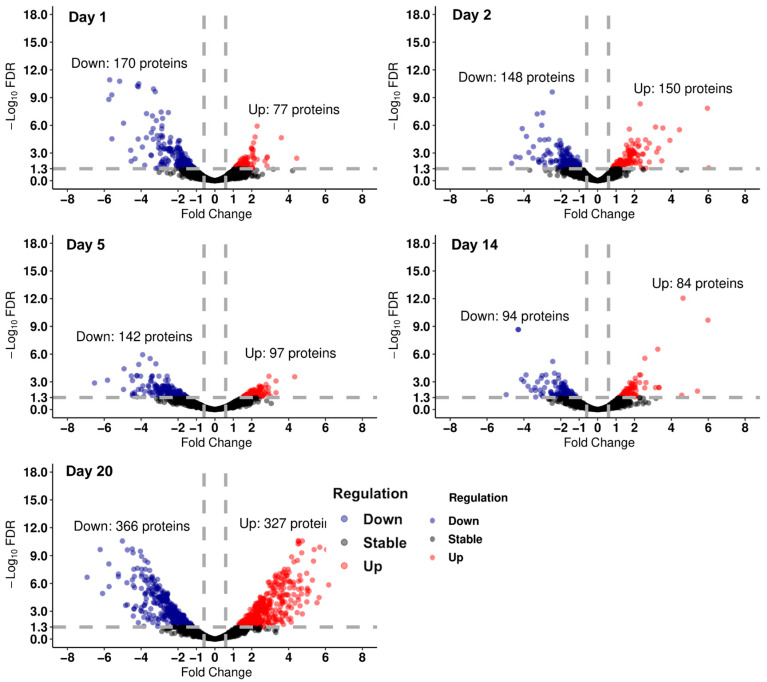
Volcano plots for differential proteins of drought group in time series. The five plots show differential abundances of proteins on days 1, 2, 5, 14, and 20, respectively. The criteria for fold change of proteins range less than log_2_(−1.5) = −0.585 and greater than log_2_(1.5) = 0.585 (left side of the left vertical dashed line and right side of the right vertical dashed line), and the criteria for −log10 FDR is 1.3 on the *y*-axis (equal and above the horizontal dashed line). The blue dots are down-regulated proteins with criteria (fold change ≤ −0.585, FDR < 0.05) and the red dots are up-regulated proteins with criteria (fold change ≥ 0.585, FDR < 0.05). The black dots are proteins that do not change (FDR > 0.05).

Compared to the control SWC on each day, we identified proteins with significantly different abundances in the drought group (Figure 4, Supplementary Dataset 1). In general, we observed a higher proportion of down-regulated than up-regulated proteins in the drought-exposed animals (Figure 4). On the last day of the drought experiment, day 20, we observed a major response in the springtail proteome for the drought group, involving 366 up-regulated proteins and 327 down-regulated proteins (Figure 4).

Among the most notable drought-responsive proteins, we identified two types of peritrophin and four types of chitinases in the proteomic data of *F. candida* (Supplementary Dataset 1), which was probably related to the cessation of growth in dry soil.

Vitellogenin proteins, a family of glycolipoproteins that are precursors to the egg yolk proteins found in maturing eggs, were also responsive to soil moisture and down-regulated in dry soil (Supplementary Dataset 1).

We identified four heat shock proteins (HSPs) in this study, but no clear pattern occurred pointing towards a general stress response prompted by exposure to dry soil (Supplementary Dataset 1).

**Figure 5:**
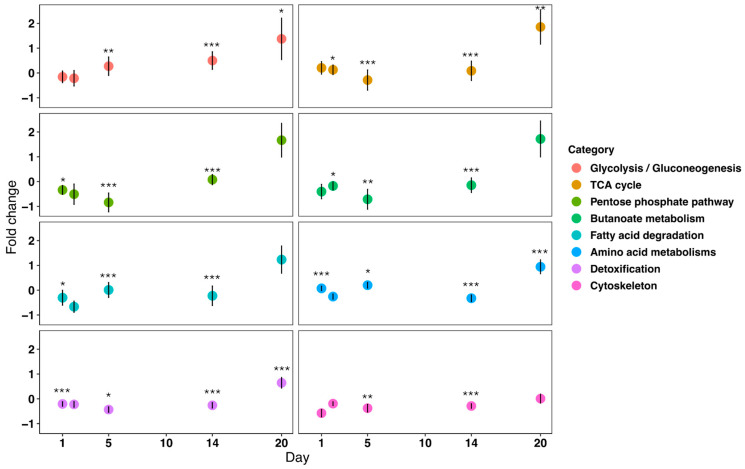
Changes in metabolic pathways in *Folsomia candida* under drought stress from day 1 to 20 with 8 metabolic pathways shown in different colors on days 1, 2, 5, 14, and 20. Each dot shows mean values of the pathway fold change each day. Black bars are standard deviations for fold change derived from differentially abundant proteins measured in each pathway (minimum 7 up to 157 proteins in a pathway). The asterisks represent significant fold changes in a pathway, compared to the control on specific days (“*” for *p* < 0.05, “**” for *p* < 0.01, “***” for *p* < 0.001).

The identified proteins were mainly distributed among 31 pathways out of 119 known for *F. candida* in the KEGG database (Supplementary Dataset 2). In particular, responsive pathways were significantly enriched under drought stress at different time points (Figure 5, FDR < 0.05, Supplementary Dataset 3). Six pathways initially decreased (day 1 to 5) and then significantly increased up to more than five times higher than in controls from then until day 20 (Figure 5), including glycolysis/gluconeogenesis from −0.16 to 1.4 fold changes (FDR < 0.01), TCA cycle from 0.2 to 1.9 fold changes (FDR < 0.01), pentose phosphate pathway from −0.3 to 1.7 fold changes (FDR < 0.01), butanoate metabolism from −0.4 to 1.7 fold changes (FDR < 0.01), fatty acid degradation from −0.3 to 1.2 fold changes (FDR < 0.01), and amino acid metabolisms from 0.07 to 0.94 fold change (FDR < 0.01). Additionally, the cytoskeleton pathway slightly changed from −0.58 to 0.01 fold change (FDR < 0.01) (Figure 5). Detoxification pathways were, in general, down-regulated in the beginning of the drought exposure but significantly raised at day 20 (FDR < 0.01). However, Glutathione S-transferase, Glutathione S-transferase theta-1, and Glutathione S-transferase 5—enzymes which are involved in Glutathione metabolism for detoxification and oxidative stress elimination—showed significantly increased fold changes of 2.1, 4.7, and 2.5, respectively, under drought stress on days 2, 14, and 20 (Supplementary Dataset 3).

In the Glycolysis/Gluconeogenesis and the TCA cycle, a dozen key enzymes were identified and enriched, and their fold changes were impacted by drought and/or the interaction effect of drought and time (Figure 4 and Supplementary Dataset 4). For example, there was an interaction effect of drought and time on the rate-limiting enzyme Fructose-bisphosphate aldolase (FBA), which was significantly up-regulated from −2.3 to 4.5 fold changes from day 2 to 20 (FDR < 0.01), but not by either drought or time (FDR > 0.05). Moreover, another interaction effect of drought and time on aldehyde dehydrogenase (ALDH) was detected, where the fold change decreased from 1.6 to −2.3 from day 5 to 20 (FDR < 0.01), suggesting probably ethanol accumulated due to the up-regulated alcohol dehydrogenase (ADH), instead of acetate (Figure 4). Although we did not detect more interaction effects on other enzymes in glycolysis, their changes are important to the sugar accumulations at low soil moisture.

**Figure 6:**
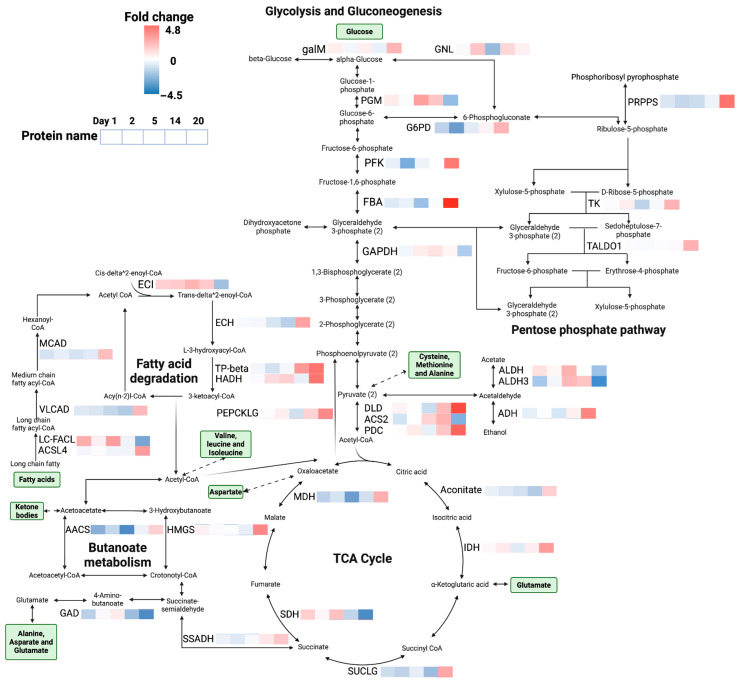
Responsive pathways of *Folsomia candida* exposed to drought. Five essential pathways in this network are shown, including glycolysis/gluconeogenesis, TCA cycle, pentose phosphate pathway, butanoate metabolism, and fatty acid degradation. The five horizontally connected squares next to the protein name represent days 1, 2, 5, 14, and 20, respectively, and different colors in each square show the fold changes of up-regulated or down-regulated proteins in the pathways on each day, ranging from blue to red (−4.5 to 4.5). The green boxes indicate the names of carbohydrates, amino acids, and sugars in the pathways. Unidirectional and bidirectional arrows represent the steps as irreversible or reversible, respectively. The dashed arrows represent multiple steps in these biochemical processes.

Enzymes for the metabolism of long-chain fatty acids were detected (Figure 6). Most were up-regulated in the drought group compared to the control group (Supplementary Dataset 4). An interaction effect of drought and exposure time on the trifunctional enzyme beta subunit (TP-beta) showed that the fold changes in TP-beta increased from −1.5 to 3.6 from day 2 to 20 (FDR < 0.01), along with effects by drought or/and exposure time (FDR < 0.01). Medium-chain specific acyl-CoA dehydrogenase increased from −1.4 to 3.6 with the effect of exposure time from day 1 to 20 (FDR < 0.05).

We detected interaction effects of drought and exposure time for enzymes in amino acid metabolisms in this study (Figure 6 and Supplementary Dataset 4). For example, S-methyl-5′-thioadenosine phosphorylase (MTAP) in cysteine and methionine biosynthesis increased from −4.2 to 1.1 fold changes with an effect from drought stress (FDR < 0.05) and an interaction effect of drought and time from day 1 to 20 (FDR < 0.01). Amidophosphoribosyl transferase (PPAT) increased from −1.8 to 2.4 fold changes with an interaction effect of drought and time from day 1 to 20 (FDR < 0.05). More interaction effects of drought and time were detected in the amino acid metabolism, playing important roles in other metabolic pathways, such as Acetoacetyl-CoA synthetase (AACS) in butanoate metabolism that increased from −2.4 to 1.5 fold changes (FDR < 0.01), also impacted by drought (FDR < 0.01) and exposure time (FDR < 0.01), and TP-beta in fatty acid degradation as well.

In detoxification pathways, we mainly identified enzymes in Glutathione metabolism (Supplementary Dataset 4). There was an interaction effect of drought and exposure time from day 1 to 20 on Glutathione S-transferase theta-1 (GSTT1), which increased from −2.8 to 4.7 fold changes (FDR < 0.01). Glutathione S-transferase 1, isoform D (GSTD1), was also significantly impacted by drought and the interaction effect, which increased from −2.1 fold changes on day 1 to 0.3 on day 14 (no data on day 20). Moreover, Gamma-glutamyltranspeptidase 1(GGT) was down-regulated by −1.9 on day 14 and −1.8 on day 20. Glutathione S-transferase Mu 1 (GSTM1) and 5 (GSTM5) showed −2.1 fold changes on day 2. Glutathione S-transferase 1 (GST) showed −3.2 fold changes on day 20. The FC for ornithine decarboxylase (OAZ) was 2.7 on day 20. 1-Cys peroxiredoxin (1-Cys Prx) had −1.7 fold changes on day 5 and 1.9 on day 20. Microsomal Glutathione S-transferase (MGST) showed −2 fold changes on day 5 and 4.5 on day 20. 5-oxoprolinase (OPLAH) showed −1.7 fold changes on day 14 and 3.4 on day 20. Glutamate–cysteine ligase (GSHA) showed 2.5 fold changes on day 20.

## 4. Discussion

The general proteomic response of *F. candida* when exposed to dry soil was an initial down-regulation of essential pathways related to metabolism and growth. After a depression during the first 5 days, these pathways gradually returned to the same levels as in moist soil. This pattern likely reflects the quick drop in body water content when springtails were transferred to dry soil, followed by a slow increase in hydration until animals, after two weeks, reached the same body water content as the animals in moist soil. Soil invertebrates may respond to dry conditions by becoming less active, or even enter dormancy, characterized by depressed respiratory metabolism [34,35,36]. Moreover, we observed that animals in the dry soil did not increase their body mass, which is consistent with the depressed metabolic pathways in the first part of the drought exposure.

Drought stress poses a threat to the survival, growth, and reproduction of *F. candida* [15]. These springtails have evolved the ability to tolerate drought stress by accumulating compatible osmolytes that increase body fluid osmolality, thereby countering body water loss and even facilitating water vapor absorption across the skin [16]. A transcriptomic study demonstrated that differentially expressed transcripts of *F. candida* under drought stress are involved in diverse biological processes, such as the glucose catabolic process, carbohydrate transport, the chitin metabolic process, oxidative phosphorylation, and the juvenile hormone metabolic process [23].

In the present study, the proteome did not show a dramatic and rapid induction of proteins in response to drought stress when the body water content dropped. For example, on day 1, there were twice as many down-regulated proteins as up-regulated proteins. This result contrasts with the study by [23], which reported positive and rapid transcriptomic responses to counter drought conditions (relative humidity 98.2%) after 24 h. Altered protein abundance for cellular functions occurs downstream of gene expression and hence displays a delayed response to the environmental changes compared to transcriptomic levels. Moreover, other studies, such as those by [37], show that molecular responses in organisms can be disparate between gene and protein and are complex due to temporal constraints and multi-dimensional regulation (i.e., microRNA, post-transcriptional, post-translational modifications of proteins, etc.)

Although the immediate overall responses of the proteome were not as expected based on earlier reports on transcriptomic responses to drought [23], the changes in proteins and pathways related to the accumulation of desiccation-protective compatible osmolytes were time-dependent, suggesting that after an initial decrease, these pathways became highly active later (at day 20), where water balance was re-established. Butanoate metabolism and pentose phosphate metabolism became carbon stores for fatty acid degradation and glycolysis, respectively. The ethanol pathway, namely glycolysis and ethanol production, contributed to sugar alcohols for osmoregulation. Noteworthy was that no significant changes in the respiratory chain were detected over the drought exposure. This is hypothesized to be due to accumulated carbohydrates for springtails under drought stress, rather than complete catalysis to the final product CO_2_. Here, the results suggest that the transformation of fatty acids to sugars increased the osmolality of the animal hemolymph, facilitating water vapor absorption.

Several other pathways were affected by drought over time. For example, the cytoskeleton pathway, which is important in cell proliferation and differentiation [38], was predominantly down-regulated throughout the experiment in drought-exposed animals, which correlates with absent or negligible somatic growth of the springtails. In addition, the early responses of several enzymes in the beta-oxidation of unsaturated fatty acids and pentose pathways suggested that fatty acid degradation and carbohydrate synthesis were initiated in the first couple of days. This may relate to an altered composition of membrane phospholipid fatty acids of drought-exposed *F. candida* [39]. Detoxification, especially glutathione metabolism, also increased with the increasing beta-oxidation of fatty acids. Moreover, the observed decrease in peritrophin suggests a loss of structural and functional integrity of peritrophic membranes composed of chitin and protein, and the increase in chitinases indicates the breakdown of chitin structure, likely facilitating water absorption by balancing osmolality through thin layers of chitin skeletons and loss of structure of peritrophin within 5–14 days under drought stress. When the body water content in animals from the drought group became higher than the control, peritrophic proteins increased and chitinases decreased on day 20.

In summary, our results suggest that the proteomic response of *F. candida* to drought stress is highly time-dependent. Proteins in pathways related to carbon flux from fatty acids to sugars increased during the exposure to dry soil, and this may relate to gradual body water content increases over time, which eventually reached the same level of hydration as in control animals exposed to moist soil. Furthermore, proteins related to growth were inhibited, and proteins in reproduction were significantly down-regulated. This study contributes to understanding mechanisms related to drought tolerance in springtails and other arthropods. Increased knowledge of the capacities of soil arthropods to tolerate increased intensity and frequency of dry periods is important in order to understand the consequences of climate change for soil functioning and fertility.

## Data Availability

The data presented in this study are available on request from the corresponding author due to restrictions.

## Supplementary Materials

The following supporting information can be downloaded at https://www.mdpi.com/article/10.3390/insects16070707/s1, Supplementary Figure S1. Distribution of protein abundances and qualities. Supplementary Figure S2. Interaction effect of drought and time on *Folsomia candida*. Supplementary Dataset 1. Fold changes of proteins at five time points. Supplementary Dataset 2. Enriched pathways in *Folsomia candida*. Supplementary Dataset 3. Significantly responsive pathways in *Folsomia candida*. Supplementary Dataset 4. Interaction effect of drought and time on proteins in pathways.
