# Supplementary material for: Proteomic Responses of the Springtail Folsomia candida to Drought
Source: Insects. 2025 Jul 9;16(7):707. doi: 10.3390/insects16070707 (PMC12295829; doi:10.3390/insects16070707)
Supplement: Supplementary file 1 [file insects-16-00707-s001.zip › Supplementary Figures.pdf]

Supplementary Figures for

Proteomic responses of springtails to drought

Authors: Yang Wang[1], Stine Slotsbo[1], Steffen Y. Bak[2], Christopher J. Martyniuk[3], Martin Holmstrup[1]*

[1] Department of Ecoscience, Aarhus University, C.F. Møllers Allé 4, 8000 Aarhus C, Denmark

[2] International Flavors & Fragrances Inc., Edwin Rahrs Vej 38, 8220 Brabrand, Denmark

[3] Center for Environmental and Human Toxicology, University of Florida, 2187 Mowry Road, Gainesville, FL 32611, USA

*Corresponding author: martin.holmstrup@ecos.au.dk

Summary:

This file has 2 supplementary figures in 3 pages

Supplementary Figure S1. Distribution of protein abundances and qualities.

Supplementary Figure S2. Venn diagram of protein numbers response to drought duration, low soil water content and the factors' interaction.

More 4 supplementary datasets are NOT included in this file.

Supplementary Dataset S1. Fold changes of proteins at 5-time point.

Supplementary Dataset S2. Enriched pathways in *Folsomia candida.*

Supplementary Dataset S3. Significantly responsive pathways in *Folsomia candida.*

Supplementary Dataset S4. Interaction effect of drought and time on proteins in pathways.

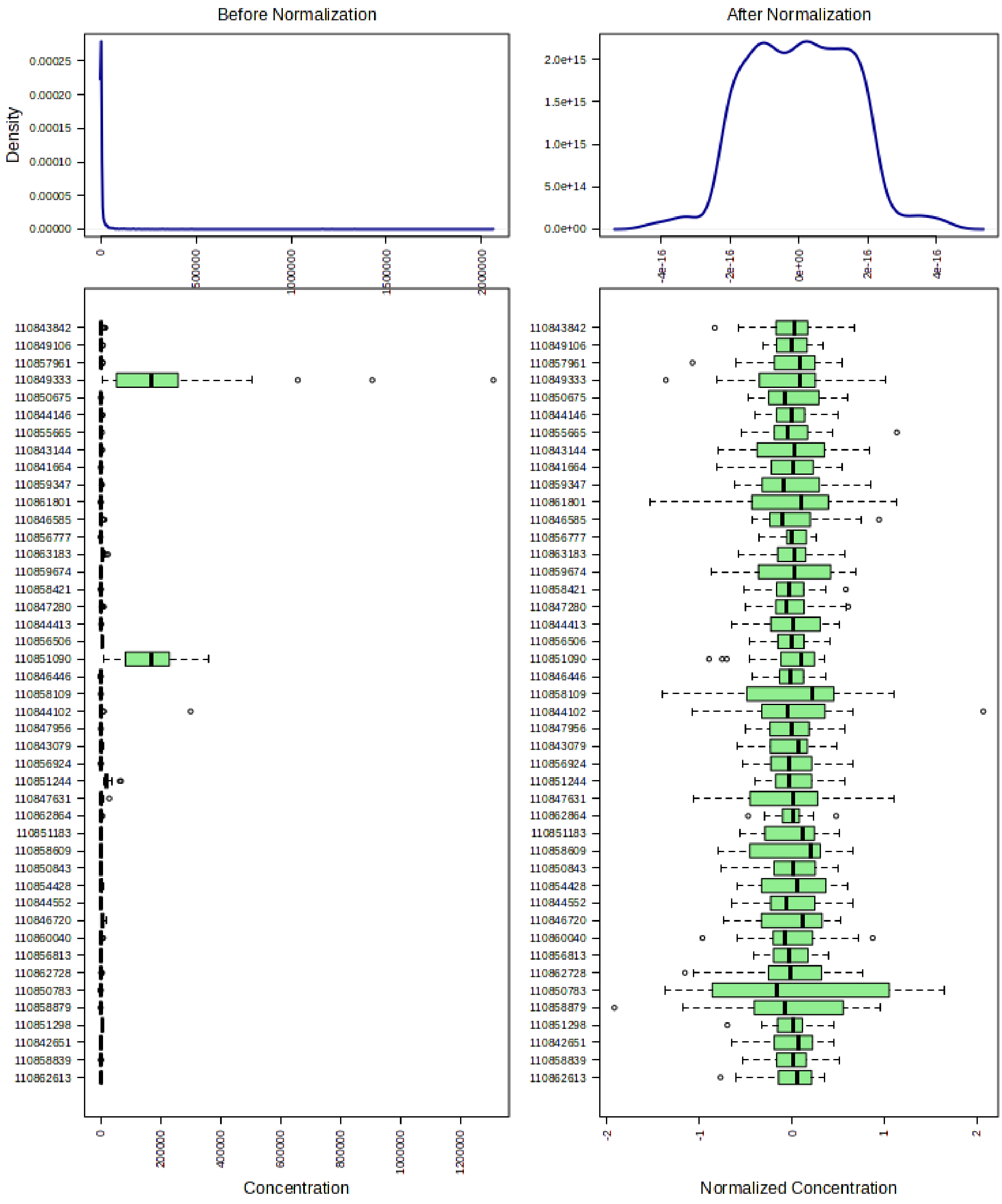

Supplementary Figure S1 Distribution of protein abundances and qualities. The two diagrams on the left side show the distribution of protein abundances and the diagrams on the right side show the distribution of normalized protein abundances (44 proteins as examples from total proteins)

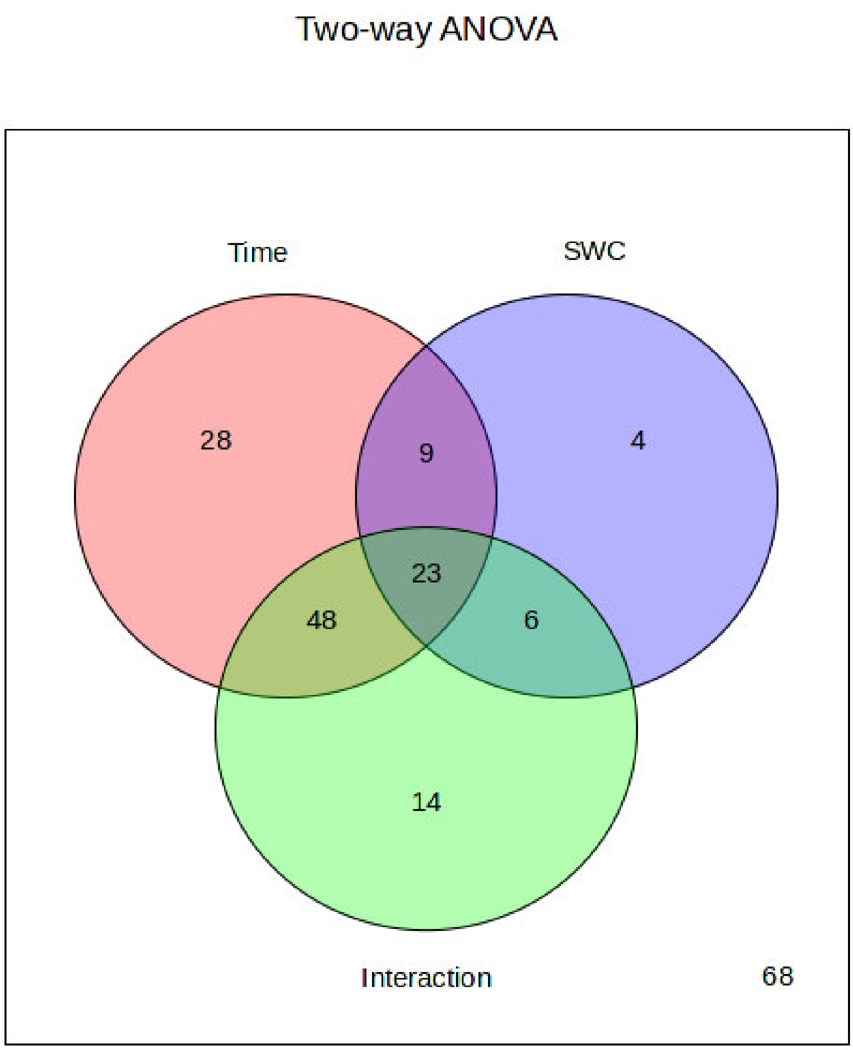

Supplementary Figure S2 Venn diagram of protein numbers response to drought duration, low soil water content and the factors' interaction. The pink circle shows that the numbers of proteins are impacted by drought duration, including 28 unique proteins only for time. The purple circle shows that the numbers of proteins are impacted by low soil water content, including 4 unique proteins only for low soil water content. The green circle shows the numbers of proteins are impacted by the interaction, including 14 unique proteins only for the interaction. The numbers in the overlapped areas mean the numbers of proteins are impacted by the two factors or each of them conbined with the interaction, including 23 proteins responses to both factors and their interaction. Total 68 proteins are not impacted by the factors and their interaction.
